# Personality Matters: Owner‐Ascribed Personality Predicts Range Size Tendencies and Predation Behavior in Domestic Cats

**DOI:** 10.1002/ece3.73520

**Published:** 2026-04-29

**Authors:** Bastien Berrand, Emmanuelle Baudry, Elsa Bonnaud

**Affiliations:** ^1^ Université Paris Saclay, CNRS, AgroParisTech, Ecologie Société Evolution Gif‐sur‐Yvette France; ^2^ Université de Lorraine, INRAE, Laboratoire Animal et Agroécosystèmes Nancy France

**Keywords:** animal‐borne cameras, behavioral ecology, domestic cats, personality, predation behavior, space use

## Abstract

Free‐ranging domestic cats ( *Felis catus* ) are recognized as invasive and efficient predators on islands, although their impact on continental wildlife remains poorly understood. Predation behavior and space use are key indicators for estimating the influence of these felines on prey populations through direct killings and sublethal effects (landscape of fear). Previous work highlighted—and debated—the importance of biological (sex, age) and environmental (habitat type) factors as well as owner‐related habits (feeding, play, hygiene) in shaping hunting and roaming behaviors. However, the influence of individual behavioral characteristics, known as personality, remains largely unexplored. In this study conducted in a suburban area south of Paris, we monitored 23 domestic cats using combined GPS and animal‐borne camera devices (or “kittycams”) between March and May 2025. Cat predation behavior was analyzed using a detailed ethogram, and space use was studied through two parameters: core range (aKDEc50) and full range (aKDEc95). Although additional data would certainly strengthen our conclusions, our results already reveal that personality affects both range size and hunting propensity. More precisely, cats with high levels of agreeableness and neuroticism have smaller range sizes and hunt less than other individuals. Moreover, animal‐borne cameras are far more reliable than the prey‐report method to assess predation events and prey diversity, as only one prey was discovered of the 31 caught in total. These results pave the way for tailored, effective, and ethical management measures to mitigate the impact of domestic cats on wildlife.

## Graphical abstract

Free‐ranging domestic cats ( *Felis catus* ) can impact wildlife through predation and landscape of fear. We monitored 23 suburban cats south of Paris using GPS collars and animal‐borne cameras and assessed personality traits via owner questionnaires. Results show that personality influences home range size tendencies and hunting behavior, providing insights for understanding and managing the ecological impact of domestic cats.

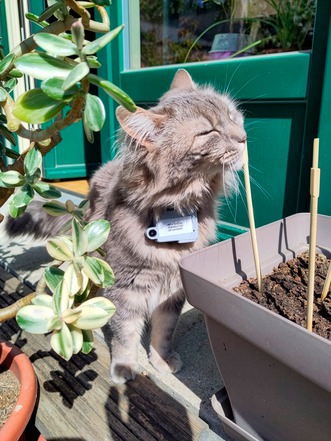

## 1 Introduction

Through various mechanisms, including the domestication of animals and the introduction of exotic species, humanity has profoundly reshaped biocenoses (Svenning et al. 2024). The numerous episodes of domestication correspond to the needs of human populations to control the services provided by other species (e.g., source of food, labor, transportation, companionship) (Driscoll, McDonald, and O'Brien 2007; Librado et al. 2021). Many domesticated taxa, ranging from dogs and livestock to cultivated ornamental plants, have spread alongside human migrations and established themselves through human activities (propagule pressure) (Driscoll, McDonald, and O'Brien 2007). These established exotic species, commonly referred to as invasive species, are now widespread and cause a range of severe damage to the ecosystems into which they have been introduced (Carneiro et al. 2026).

The domestic cat ( *Felis catus* ) embodies the duality of a domesticated species that is also invasive in some ecosystems, especially on islands, where it often becomes the top predator (Woinarski et al. 2015; Loss et al. 2022; Lepczyk et al. 2023). The first human–cat interactions beginning 10,000 years ago were likely an opportunity for cats to feed on small rodents attracted by crops rather than a deliberate form of domestication (Driscoll, Menotti‐Raymond, et al. 2007; Crowley et al. 2020). Since then, domestic cats have spread with human migration across every continent except Antarctica (Lepczyk et al. 2023). Despite their domestication, cats still retain an independent nature, and most owners allow their cats to roam freely. As obligate carnivores, cats prey on many different taxa (mainly small mammals, birds, insects, and reptiles) (Loyd et al. 2017; Cordonnier et al. 2022; Castañeda et al. 2023). Although killings obviously reduce the fitness of prey and compromise the future of the species, sublethal effects should not be underestimated. Even when they do not actively hunt, cats can still trigger vigilance behavior and stress in the prey species, which detect their predators (and clues of their presence like territorial marks or feces) by sight, smell, or sound. This phenomenon, called the “landscape of fear” (Fardell et al. 2023), has both physiological and behavioral consequences for prey, notably affecting reproduction (early reproductive senescence, shorter offspring life expectancy due to reduced parental care, smaller clutches) and foraging (Bonnington et al. 2013; Balbontín and Møller 2015; Mahlaba et al. 2017). Nonprey species can also be impacted by domestic cats in their environment through interspecific competition (Biró et al. 2005; Merson et al. 2020; Rees et al. 2023), transmission of diseases such as toxoplasmosis and feline leukemia (Loss and Marra 2017), or hybridization (Germain et al. 2008; Todesco et al. 2016).

The combination of predatory traits, roaming ability, and large numbers of individuals can have profound consequences on the ecosystem through top‐down processes. The negative impact of cats on vertebrate prey has been reported on at least 120 islands, affecting at least 175 species (25 reptiles, 123 birds, and 27 mammals), which highlights the urgent need to continue management actions to preserve the biodiversity of island endemic species (Jones et al. 2016; Brooke et al. 2018). In continental areas such as Europe, domestic cats are prevalent in agricultural and suburban areas where the human population is growing and cat densities can reach several hundreds of individuals per square kilometer (Liberg et al. 2000). They often have access to natural and semi‐natural areas, including private gardens, fields, meadows, and small woodlands, suggesting their high potential impact on the fauna of these habitats, especially birds (Woods et al. 2003; Pavisse and Vangeluwe 2019). However, the effects are probably weaker than on islands because they are native predators that have evolved alongside their prey (Doherty et al. 2016). Although cats can exert high predation pressure, their subsequent impact on prey population dynamics may be limited if predation is concentrated on abundant species (Philippe‐Lesaffre et al. 2025). Overall, if the impact of domestic cats on wildlife in suburban areas is acknowledged, it remains poorly quantified, even as their numbers increase alongside ongoing urban sprawl (Aegerter et al. 2017; Philippe‐Lesaffre et al. 2024).

To assess the impact of domestic cat on prey species, several parameters can be monitored. Predations events are obviously an important one, but sublethal effects induced by the landscape of fear can only be accessed through indirect measures, like the range size (Philippe‐Lesaffre et al. 2024). Studies report varying estimates of the average range area of pet cats, which generally falls between 1 and 4 ha around the house (Thomas et al. 2014; Hanmer et al. 2017; Kays et al. 2020; Cecchetti, Crowley, Wilson‐Aggarwal, et al. 2022; Philippe‐Lesaffre et al. 2024). Several parameters can influence the range size. For example, it tends to decrease along an urbanization gradient (Hanmer et al. 2017; Kays et al. 2020; Cordonnier et al. 2022), with larger ranges observed in suburban and rural settings, in part due to human infrastructure such as buildings, fences, barriers, and roads, which can significantly restrict cats' natural range and mobility in urbanized areas (Thomas et al. 2014). Sex, age, and roaming restrictions are commonly discussed factors influencing range size, although no consensus has yet emerged (Thomas et al. 2014; Hanmer et al. 2017; Kays et al. 2020; Cecchetti, Crowley, Wilson‐Aggarwal, et al. 2022; Philippe‐Lesaffre et al. 2024). Seasonal and circadian variations appear to be negligible (Thomas et al. 2014; Hanmer et al. 2017; Philippe‐Lesaffre et al. 2024). Predation behavior is also influenced by multiple factors. The size and composition of the home range affect predation, with cats that have larger ranges tending to bring home a more diverse prey assemblage, as they explore more diverse habitats and are generalist hunters (Loyd et al. 2013; Krauze‐Gryz et al. 2017; Hernandez et al. 2018; Cordonnier et al. 2022). In urban areas, the density of some commensal species such as the brown rat ( *Rattus norvegicus* ) is usually high, but the diversity of species is low, meaning that cats do not usually prey on threatened or rare species (Herrera et al. 2022). However, the risk increases closer to natural habitats such as forests (Herrera et al. 2022). The type of habitat also influences the kill rate: The kill rate of birds increases with the presence of trees (Cordonnier et al. 2022), whereas that of small rodents and reptiles increases in open lands (McGregor et al. 2015). The relation between sex, age, coat colors/patterns and predation behavior is subject to debate (Mella‐Méndez et al. 2022; Castañeda et al. 2023; Cordonnier et al. 2022, 2023). Owners' practices through feeding, play, and hygiene can affect hunting behavior. Low protein food tends to increase predation, as cats compensate by hunting wild prey to meet their nutritional needs (Cecchetti, Crowley, and McDonald 2021; Herrera et al. 2022). Playing with cats can reduce predation, although this effect appears to be limited to mammalian prey (Cecchetti, Crowley, and McDonald 2021).

Even after accounting for environmental and biological factors, as well as owners' practices, substantial interindividual variability remains. This variability is likely driven by individual behavioral traits, also known as personality (Cecchetti, Crowley, McDonald, and McDonald 2022; Philippe‐Lesaffre et al. 2024; Cordonnier et al. 2023, 2022). Animal personality, also referred as temperament or behavioral syndromes (Litchfield et al. 2017), is defined as “consistent individual differences in behavioral patterns” (Koski 2014; Litchfield et al. 2017). Long disregarded, animal personality is now an emerging subject of study, with a growing interest in its influence on ecological processes (Réale et al. 2010; Sih et al. 2012; Wolf and Weissing 2012; Roche et al. 2016). Pets are well suited to this study, as their owners can accurately evaluate their personalities (Gosling et al. 2003). A widely used reference for assessing the personality of domestic cats is a test developed by Litchfield et al. (2017) based on questionnaires completed by owners of free‐ranging cats around the “feline five” traits: agreeableness, dominance, extraversion, impulsiveness, and neuroticism. To date, no study has directly linked cat personality to range size, which represents the extent of their influence through the landscape of fear. Some studies have already investigated the relationship between personality and predation, demonstrating higher predation rates for cats with high levels of extraversion and dominance and low levels of neuroticism and agreeableness (Cecchetti, Crowley, McDonald, and McDonald 2022; Cordonnier et al. 2023).

However, these studies employ the prey report method (declaration of the quarries found by the owners), which is useful but biased, as it only accounts for about one‐third of prey catches by cats (Loyd et al. 2017; Cordonnier et al. 2023). Animal‐borne cameras (or “kittycams”) thus represent a more accurate method of study (Loyd et al. 2013; McGregor et al. 2015; Hernandez et al. 2018; Huck and Watson 2019; Seymour et al. 2020) and are also easily applicable to pet cats as most of them tolerate wearing a collar (Lord et al. 2010). This technique has yet to be applied to the study of the relation between cat personality and hunting behavior.

This study investigates whether the personality of domestic cats influences their roaming and predatory behaviors by fitting 23 suburban domestic cats with innovative devices combining GPS and cameras. Only diurnal behaviors were monitored as birds and herpetofauna were the main focus and are more targeted during the day (Woods et al. 2003). This was also more adapted to our study area, where a significant proportion of owners lock their cats indoors at night for various reasons, including protecting biodiversity and ensuring animal safety. The data collection took place from March to May, coinciding with the period of higher predation on birds and herpetofauna (Castañeda et al. 2023). Based on the existing body of literature, we predict that cats with high levels of extraversion and dominance and low levels of neuroticism and agreeableness have a larger range and hunt more frequently (Cecchetti, Crowley, McDonald, and McDonald 2022; Cordonnier et al. 2023), with small mammals and birds constituting a significant proportion of their prey (Krauze‐Gryz et al. 2017; Castañeda et al. 2023). These results would enable stakeholders to implement targeted measures to mitigate the impact of each cat while ensuring its well‐being.

## 2 Material and Methods

### 2.1 Ethics Statement

The entire study complied with French legislation. All cat owners voluntarily participated in the study by fitting collars to their cats and answering questionnaires. All participants were given a General Data Protection Regulation document and provided written informed consent. All necessary precautions were taken at each stage of the study to ensure the welfare of the cats (use of breakaway collars, acclimatization phase…) and procedures implemented during this study were strictly noninvasive. Consequently, as defined in Article 1(5)(f) of Directive 2010/63/EU, no formal approval from an animal ethics committee was needed.

### 2.2 Study Area and Cat Recruitment

**FIGURE 1:**
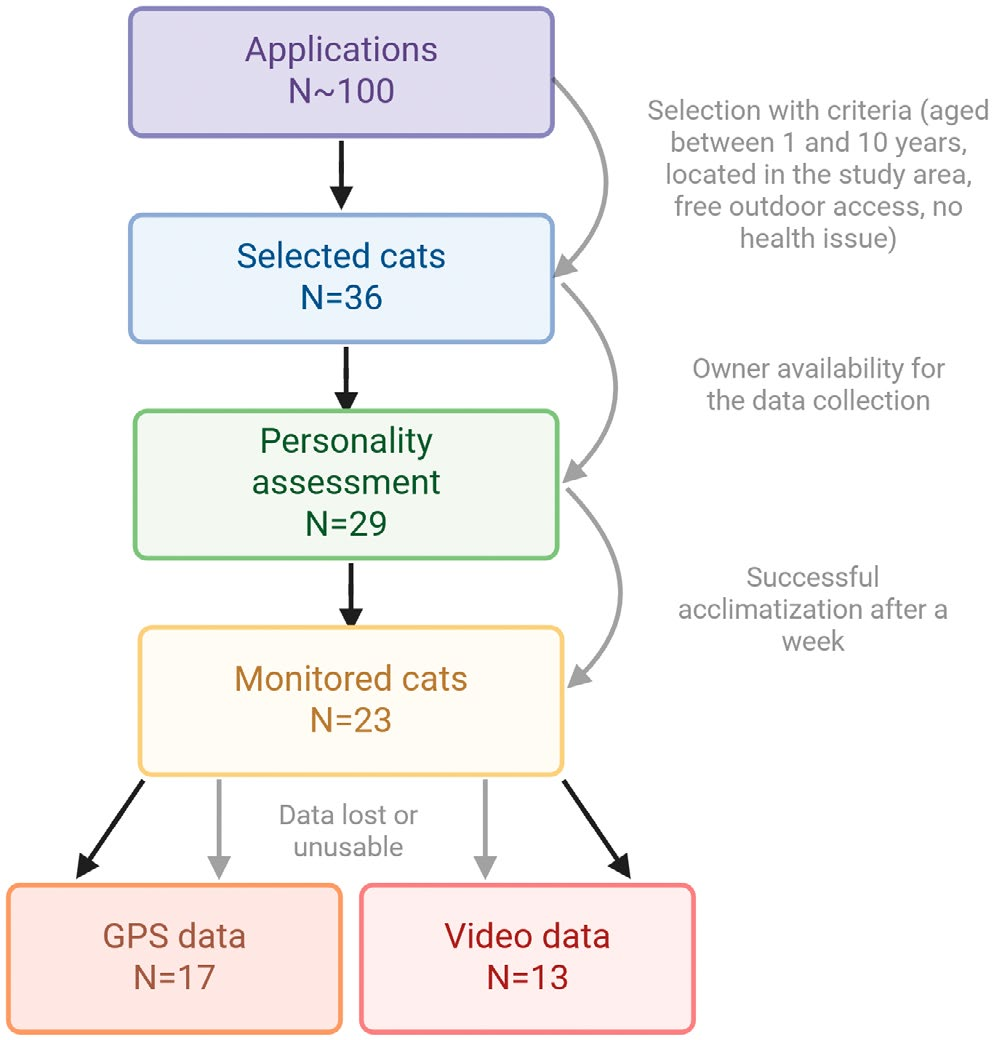
Number of cats at each step of the protocol.

The study took place in the lower part of the Chevreuse Valley in southern Paris, France. This suburban area consists mainly of single‐family housing developments, crisscrossed by agricultural land (mostly cereal crops) and small wooded areas (hardwood). Thirty‐six cats were recruited (Figure 1) through local and online communication based on the following selection criteria: (i) the cat's home located in the study area; (ii) age between 1 and 10 years; and (iii) outdoor access via a cat flap. The cat flap ensured that the cats could come and go as they pleased, so their tendency to spend time outdoors was not influenced by their owners. Cats aged less than 1 year were excluded from the study because their personalities had not yet formed, and the recording devices were too heavy for them to carry. Cats older than 10 or 11 years were also excluded as they tend to be less active due to their age. Cats' health was also carefully considered, and one cat was excluded from the study because it was polydactyl and could not climb fences. One cat was excluded after 4 days due to a leg injury (unrelated to our protocol). All cats were sterilized and crossbreed (also known as “European”). Seven cats were finally excluded from the study due to their owners' unavailability, leading to the inclusion of 29 cats.

### 2.3 Data Collection and Processing

#### 2.3.1 Personality and Habit Assessment

Personality was assessed using a questionnaire for the 29 cats. Owners rated the degree to which 16 adjectives matched their cat's personality on a scale from 1 to 5 (where 1 means “not at all” and 5 means “completely”). These adjectives taken from Litchfield et al. (2017) were selected due to their strong association with four of the “feline five” traits (agreeableness, dominance, extraversion, and neuroticism) as well as their straightforward and unambiguous translation in French. Adjectives relating to the fifth trait, impulsiveness, were not included, as this trait was not consistently identified in other studies on cat personality (Cordonnier et al. 2023). Therefore, the four assessed behavioral traits and their associated adjectives were: extraversion (smart, curious, active, inventive), dominance (aggressive with other cats, brutal, irritable, dominant), agreeableness (affectionate, cuddly, gentle, friendly to people), and neuroticism (worried, anxious, shy, fearful of other cats). The “feline five” test has been used in several previous studies (Cecchetti, Crowley, McDonald, and McDonald 2022; Cordonnier et al. 2023), underlying its reliability. To conduct a robust exploratory factor analysis to link adjectives to personality traits, the results for the 29 cats of the study were merged to an existing dataset (not yet published) of 1010 other personality profiles obtained through the same questionnaire.

#### 2.3.2 Spatial and Behavioral Data From GPS/Camera Devices

**FIGURE 2:**
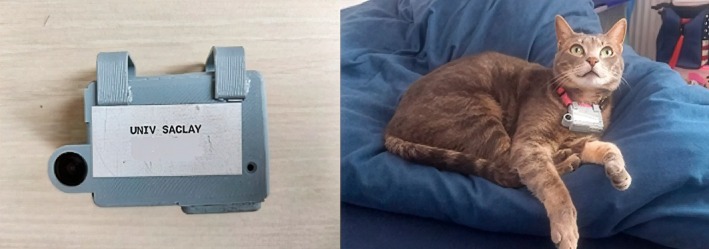
Close‐up of the device (left) and cat wearing the device (right).

The 29 cats were equipped with fake devices attached to an antistrangulation collar for a week during an acclimatization phase aimed at ensuring both data quality and animal welfare, even though the collars weighed less than 2% of the total body mass (Coughlin and van Heezik 2015; Hanmer et al. 2017). Owners were asked to report any sign of discomfort from their cat and to immediately remove the device in this case. Six cats were thus excluded from the study, as they showed disturbed behavior. The remaining 23 cats were fitted with tailor‐made combined GPS loggers (CatLog2TM model) and camera module devices (Figure 2). The unit operated cyclically with an alternance of 5 min of filming and 1 min of GPS logging. The GPS logging frequency was chosen to match the parameters from a previous study on cat range (Philippe‐Lesaffre et al. 2024) and to allow almost continuous filming. Owners were asked to turn on the devices in the morning, collect them after 8 h (maximum filming duration allowed by the device), and recharge the batteries overnight. The data collection period started in mid‐March and ended in mid‐May, the period when prey are the most vulnerable and abundant (reproductive period) (Charmantier and Gienapp 2014; Castañeda et al. 2023). Each cat wore the device for between 15 and 32 days to ensure at least 10 days of usable data, and owners were asked to report any prey brought home during this period.

GPS data were extracted from the devices using the CatLog Control Center and exported as CSV files. Due to technical issues such as lost or damaged devices, spatial data were only available for 20 cats. To ensure the accuracy of the data, GPS points recorded with less than five different satellites as well as outliers (points located in rivers or lakes or indicating improbable travel distances given the time between locations) were removed (Philippe‐Lesaffre et al. 2024). Area‐corrected autocorrelated kernel density estimations (aKDEc) were then calculated for each cat using the *ctmm* R package (Fleming and Calabrese 2025) to determine their home range size. The code was adapted from Philippe‐Lesaffre et al. (2024) to calculate aKDEc 95% (full home range size) and aKDEc 50% (core home range size). Due to the short sampling period, the movement model did not converge for three individuals, even with a pseudo‐hierarchical restricted maximum likelihood (pHREML) correction. These individuals were thus removed from the analysis, leaving 17 cats.

Video data were stored on secure hard drives and organized by category and day. Videos lasting less than 10 s and those taken inside a building were deleted. The remaining videos were analyzed using BORIS software (Friard and Gamba 2016) following an ethogram divided into four categories: exploration, predation, observation, and inactivity (see Appendix S1). Each item was a state event with a start and end time, enabling the duration of each action to be determined. The results were then exported as a CSV file, which also noted animal encounters and specific events. Predation events were defined either as a direct capture or an unsuccessful chase following a stalking phase (whether the prey was identifiable or not). It is important to observe that the recorded outdoor time is not equivalent to the total time spent outside during the day, as it depends on both the owner's availability to fit the collar and internal device factors (e.g., lag between switching on and recording, internal errors that sometimes caused the device to stop recording). Behavioral data used for this study correspond to 10 days of tracking to ensure low environmental variability. Four individuals with less than 10 days of exploitable recording were excluded from the analysis, leading to 13 monitored individuals with behavioral data.

### 2.4 Statistical Analyses

All statistical analyses were carried out using R software (version 4.4.3) (R Core Team 2025). The significance threshold was set at 0.05 (unless otherwise specified).

#### 2.4.1 Personality Traits

Explanatory factorial analysis was carried out on the 1039 cat personality test results using the *psych* package (Revelle 2025). The number of factors (traits) was fixed at four to align with the structure reported by Cordonnier et al. (2023). The threshold for factor loading significance was set to 0.4 (Hair et al. 2019).

#### 2.4.2 Effect of Personality on Home Range Size

The effect of personality on home range size was investigated for both aKDEc50 and aKDEc95. For both range sizes, Gamma‐log generalized linear models (1) were fitted to test the four personality traits as predictors (see Appendix S2). Other predictors such as age and sex could not be tested due to the low number of observations (*N* = 17). Residuals were checked using the *DHARMa* package (Hartig 2024).

(1) $$\begin{array}{c} \log\left(E\left[\text{aKDE}\right]\right)=\text{Extraversion}+\text{Agreeableness}\\ \;+\text{Dominance}+\text{Neuroticism} \end{array}$$

#### 2.4.3 Effect of Personality on Predation Behavior

Predation behavior was studied using two parameters: (i) the time spent hunting in seconds; and (ii) the amount of predation events (successful or not) (see Appendix S2). The *glmmTMB* package (McGillycuddy et al. 2025) was used to build the two models with the four personality traits as predictors (see Appendix S2). Model (i) is a Tweedie generalized linear mixed model (GLMM) with a log link, and model (ii) is a Poisson GLMM with a zero‐inflation correction. For both models, cat identity was implemented as a random factor, as each cat was followed for a period of 10 days with the time variable being included as an offset to account for the inter‐ and intraindividual difference in recording duration. Residuals were checked using the *DHARMa* package (Hartig 2024).

- $\begin{array}{c} \log\left(E\left[\text{Predation time}\right]\right)=\text{Extaversion}+\text{Agreeableness}\\ +\text{Dominance}+\text{Neuroticism}+\log\left(\text{Total time}\right)+\left(1|\mathrm{Cat}\;\text{identity}\right) \end{array}$
- $\begin{array}{c} \log\left(E\left[\text{Predation events}\right]\right)=\text{Extaversion}+\text{Agreeableness}\\ +\text{Dominance}+\text{Neuroticism}+\log\left(\text{Total time}\right)+\left(1|\mathrm{Cat}\;\text{identity}\right) \end{array}$

## 3 Results

### 3.1 Personality Scores

**FIGURE 3:**
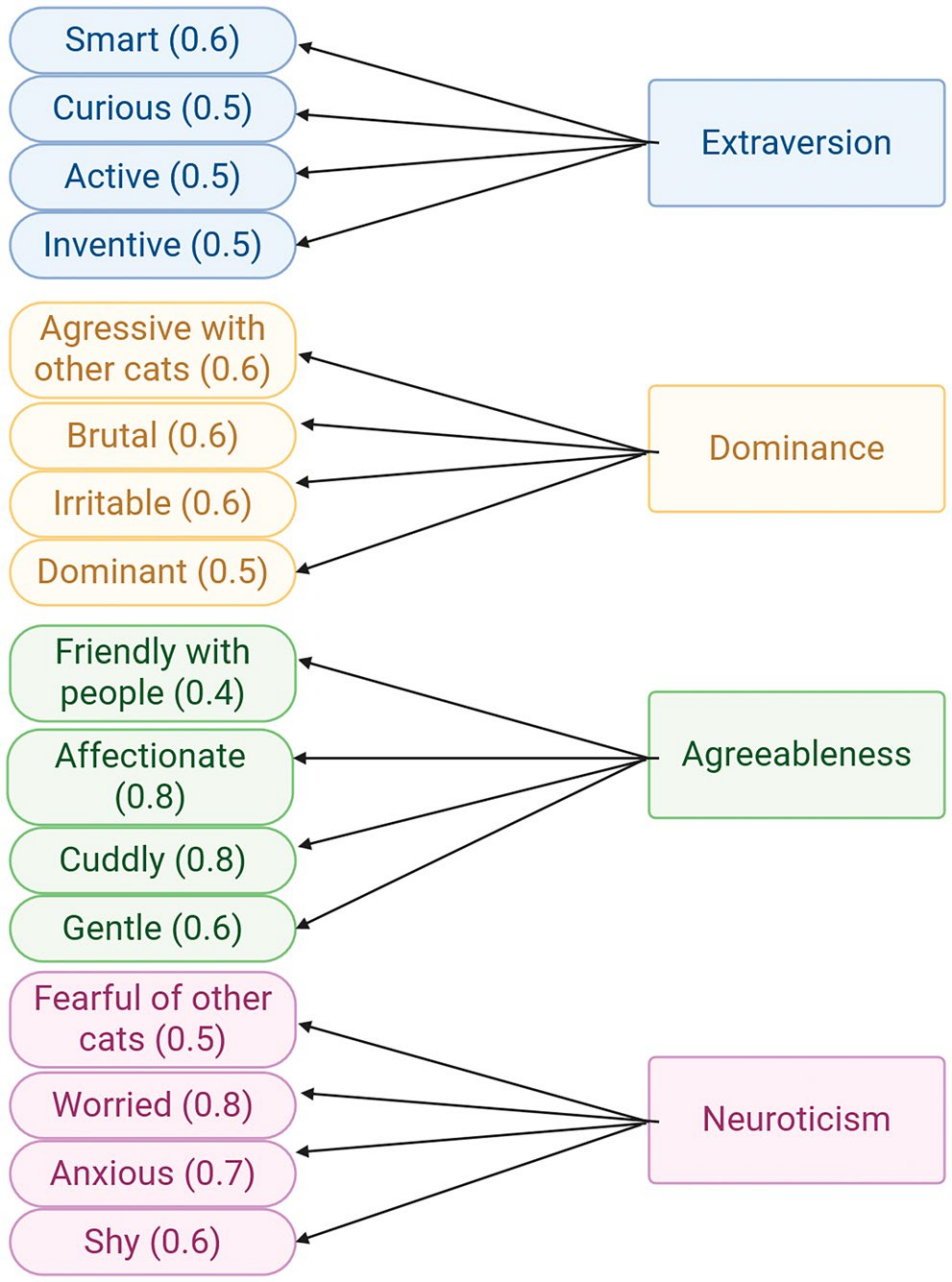
Results of the exploratory factor analysis on the cat personality tests (*N* = 1039). Values between parentheses correspond to factor loadings.

Exploratory factor analysis recovered four independent traits (agreeableness, dominance, extraversion, and neuroticism) in line with Litchfield et al. (2017) (Figure 3). Each trait is associated with four adjectives, with these associations also being consistent with (Litchfield et al. 2017). All factor loadings were equal to or superior to 0.4. The score of each cat for the four traits was then calculated.

### 3.2 Influence of Personality on Home Range Size

#### 3.2.1 aKDEc Calculation

**FIGURE 4:**
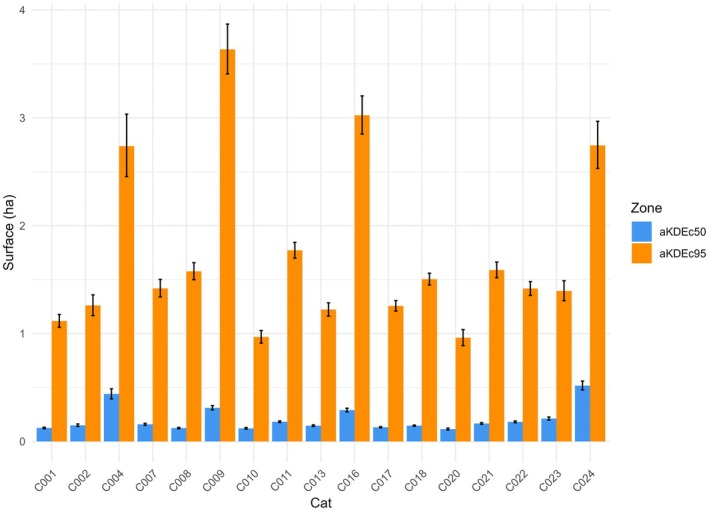
Results of aKDEc50 (core range) and aKDEc95 (full range) calculations (*N* = 17). Error bars correspond to the confidence intervals.

aKDEc50 values range from 0.11 ha to 0.52 ha, with a mean value of 0.21 ± 0.12 ha and a median value of 0.16 ha (Figure 4). aKDEc95 values range from 0.96 ha to 3.63 ha, with a mean value of 1.74 ± 0.79 ha and a median value of 1.42 ha.

#### 3.2.2 Influence of Personality on aKDEc50 and aKDEc95

For the size of the core range (aKDEc50), no personality trait had a significant effect (Table 1). Agreeableness was the only remarkable tendency (*p* = 0.051), being associated with a smaller core range size. No significant problem was detected on the residuals (Nagelkerke *R* ^2^ = 0.509).

**TABLE 1:** Summary of the aKDEc50 (core range) model.

aKDEc50 model			
Predictors	Estimates	CI	*p*
(Intercept)	0.19	0.15–0.25	< 0.001
Extraversion	1.10	0.88–1.36	0.364
Agreeableness	0.82	0.68–0.99	0.051
Dominance	0.89	0.72–1.09	0.261
Neuroticism	0.91	0.72–1.16	0.424
Observations	17		
*R* ^2^ Nagelkerke	0.509		

*Note:* log(E[aKDEc50]) = Extraversion + Agreeableness + Dominance + Neuroticism (An estimate value of 1 means no effect on the response variable).

Likewise, no particular personality trait had a significant effect on the full home range size (aKDEc95) (Table 2). Neuroticism showed a tendency (*p* = 0.062) to be associated with smaller range sizes but not significantly. No significant problem was detected on the residuals (Nagelkerke *R* ^2^ = 0.450).

**TABLE 2:** Summary of the aKDEc95 (full range) model.

aKDEc95 model			
Predictors	Estimates	CI	*p*
(Intercept)	1.67	1.37–2.07	< 0.001
Neuroticism	0.84	0.70–1.02	0.062
Extraversion	1.13	0.95–1.34	0.133
Agreeableness	0.87	0.75–1.02	0.104
Dominance	0.98	0.82–1.15	0.782
Observations	17		
*R* ^2^ Nagelkerke	0.450		

*Note:* log(E[aKDEc95]) = Extraversion + Agreeableness + Dominance + Neuroticism (An estimate value of 1 means no effect on the response variable).

### 3.3 Influence of Personality on Predation Behavior

#### 3.3.1 Behavioral Data Summary

Video data reveal that cats were mostly active (combination of exploration, predation, and observation categories) when outside during the day, with 87.7% ± 2.6% of their time spent being active on average (median = 94.5%). Of the 13 cats recorded, 9 hunted at least once during data collection, and predation events occurred on 34 of the 130 analyzed days. On hunting days, predation accounted for 2.18% ± 1.44% of their time (median = 0.39%). Interindividual and intraindividual (for one individual between days) were important.

A total of 60 predation events was recorded, with 31 prey caught and 21 prey consumed (Table 3). Predation events targeted 27 mammals (45%), 9 invertebrates (15%), 3 birds (5%), and 3 reptiles (5%) (Appendix S3). The 18 remaining prey could not be identified. The 31 captured prey included 25 mammals (80.6% of catches) as well as 3 invertebrates (9.7%), 1 bird (3.2%), and 2 reptiles (6.5%). The 21 prey items consumed by the cats included 18 mammals (85.7%) as well as 2 invertebrates (9.5%) and 1 reptile (4.8%). Only one of the prey recorded was brought home, a rodent.

**TABLE 3:** Summary of predation events, prey caught, and prey consumed.

Cat ID	Predation events						Catches					Prey consumed			
	Mammals	Birds	Reptiles	Invertebrates	Unidentified	Total	Mammals	Birds	Reptiles	Invertebrates	Total	Mammals	Reptiles	Invertebrates	Total
C002	1	1	0	0	0	2	0	0	0	0	0	0	0	0	0
C003	0	1	0	2	3	6	0	0	0	0	0	0	0	0	0
C009	18	0	0	0	6	24	17	0	0	0	17	17	0	0	17
C013	1	0	0	2	2	5	1	0	0	0	1	0	0	0	0
C015	2	0	0	0	2	4	2	0	0	0	2	0	0	0	0
C016	3	0	0	0	1	4	3	0	0	0	3	0	0	0	0
C021	0	0	0	3	0	3	0	0	0	1	1	0	0	1	1
C022	0	0	3	0	2	5	0	0	2	0	2	0	1	0	1
C024	2	1	0	2	2	7	2	1	0	2	5	1	0	1	2
Total	27	3	3	9	18	60	25	1	2	3	31	18	1	2	21

#### 3.3.2 Influence of Personality on the Time Allocated to Predation and the Amount of Predation Events

Model (i) reveals (Table 4) the significant negative effect of neuroticism (*p* = 0.035) and agreeableness (*p* < 0.001) on the time allocated to predation (in seconds). High levels of agreeableness and neuroticism were associated with shorter periods of predation. Cat identity is not negligible (ICC = 0.11) (marginal *R* ^2^ = 0.310 and conditional *R* ^2^ = 0.389). Residuals showed no deviation.

**TABLE 4:** Summary of model (i).

Model i (Predation Time)			
Predictors	Estimates	CI	*p*
(Intercept)	0	0.00–0.00	< 0.001
Neuroticism	0.34	0.13–0.92	0.035
Agreeableness	0.14	0.05–0.42	< 0.001
Dominance	1.02	0.45–2.31	0.955
Extraversion	2.16	0.92–5.03	0.075
Random effects			
*σ* ^2^	5.62		
τ00 CAT	0.73		
ICC	0.11		
NCAT	13		
Observations	130		
Marginal *R* ^2^/Conditional *R* ^2^	0.310/0.389		

*Note:* log(E[Predation time]) = Extaversion + Agreeableness + Dominance + Neuroticism + log(Total time) + (1∣Cat identity) (An estimate value of 1 means no effect on the response variable).

Model (ii) displays consistent results (Table 5) with a significant reduction in the number of predation events in agreeable cats (*p* = 0.030). The negative effect of neuroticism was not significant here (*p* = 0.087). The effect of the zero‐inflation correction is not significant (*p* = 0.102), but a likelihood ratio test showed a better fit of the model. Cat identity is not null (ICC = 0.03) (marginal *R* ^2^ = 0.048 and conditional *R* ^2^ = 0.079). No issue was detected on the residuals.

**TABLE 5:** Summary of model (ii).

Model ii (Predation events)			
Predictors	Incidence rate ratios	CI	*p*
Count model			
(Intercept)	0	0.00–0.00	< 0.001
Neuroticism	0.57	0.29–1.09	0.087
Agreeableness	0.5	0.27–0.94	0.03
Dominance	0.9	0.55–1.46	0.66
Extraversion	1.39	0.83–2.35	0.211
Zero‐inflated model			
(Intercept)	0.36	0.11–1.22	0.102
Random effects			
*σ* ^2^	9.09		
τ00 CAT	0.3		
ICC	0.03		
NCAT	13		
Observations	130		
Marginal *R* ^2^/Conditional *R* ^2^	0.048/0.079		

*Note:* log(E[Predation events]) = Extaversion + Agreeableness + Dominance + Neuroticism+log(Total time) + (1∣Cat identity) (An estimate value of 1 means no effect on the response variable).

## 4 Discussion

### 4.1 Interindividual Variation of Personality in Domestic Cats

Personality test results unveiled a large spectrum of personalities among the cats of our study. This personality is summed up by four behavioral traits: extraversion (smart, curious, active, inventive), dominance (aggressive with other cats, brutal, irritable, dominant), agreeableness (affectionate, cuddly, gentle, friendly to people), and neuroticism (worried, anxious, shy, fearful of other cats). These results are consistent with previous studies (Litchfield et al. 2017; Cecchetti, Crowley, McDonald, and McDonald 2022; Cordonnier et al. 2023) and highlight both the existence of domestic cats' personality and the reliability of the personality assessment completed by the owners.

### 4.2 Spatial Use of Suburban Domestic Cats and Influence of Personality

Cat home range sizes (here approximated by aKDEc95) range from 0.96 ha to 3.63 ha. These values are consistent with the results of several studies on cat ranges (Thomas et al. 2014; Hanmer et al. 2017; Kays et al. 2020; Cecchetti, Crowley, Wilson‐Aggarwal, et al. 2022; Philippe‐Lesaffre et al. 2024) considering our focus on a suburban area. Core range sizes (aKDEc50) do not exceed 0.52 ha. Domestic cats thus spend most of their time around the house (mainly in private gardens), undertaking explorative excursions further away in adjacent gardens, fields, or small patches of forest. This corresponds to our observations based on the video data.

The influence of personality on the home range size of domestic cats has already been hypothesized in previous studies (Philippe‐Lesaffre et al. 2024) but never directly studied to our knowledge. This study provides a deeper understanding of this influence by exploring the effects of four behavioral traits. Due to the small variability of the range sizes between individuals and the small number of individuals involved (*N* = 17), the effects of personality traits did not reach the commonly used significant threshold but nevertheless gave precious insights. For the core range, agreeableness was close to the significance threshold (*p* = 0.051) and was associated with a smaller core range. For the full range, no significant association with a behavioral trait has been found. Neuroticism was the closest to the significance threshold (*p* = 0.062), with neurotic cats tending to have a smaller range size. Personality thus influences the core range size and probably the full range size. It is assumed that these effects would be stronger with the recruitment of more participants. The results are also consistent with our hypotheses. Cats with high levels of neuroticism tend to be afraid of new situations and thus be uneasy outdoors. They remain confined to smaller areas, as they are unlikely to explore the environment far from their “safe place.” The association between shyness (here included in neuroticism) and smaller ranges was previously highlighted in other mammalian species such as the bank vole ( *Myodes glareolus* ) (Schirmer et al. 2019). Agreeable cats, which are closer to humans, also have smaller ranges. The effect is more pronounced for the core range, perhaps reflecting a tendency to remain close to their owners when they are home and exploring further afield when they are absent. Agreeableness is centered on affection toward humans, thus making it difficult to compare these results to other species as most other pets do not roam free.

Contrary to our assumptions, extraversion and dominance were not the most determinant personality traits on range size. Nevertheless, they probably influence range but their effects are not strong enough to be detected in this study. Cats with high levels of extraversion are likely to explore wider ranges as this trait is defined by attributes like curious and active. The association between extraversion (also referred to as boldness) and wider ranges was also observed in other species such as the golden‐mantled ground squirrel ( *Callospermophilus lateralis* ) (Aliperti et al. 2021). We expected dominant cats to explore wider ranges, although recent literature on Darwin's finch ( *Camarhynchus pauper* & *Geospiza fuliginosa* ) showed that aggressive individuals have smaller territories as they occupy higher‐quality areas (García‐Loor et al. 2025). For domestic cats, the house represents a high‐quality habitat as their owners provide food, water, and shelter among others. Dominant individuals would thus defend the area close to their home (including the garden) and focus their surveillance on a smaller area compared to less dominant cats. Our study recorded fights between cats of different households, all of which took place in the garden around the home of one of the cats involved, thus supporting this hypothesis.

### 4.3 Cat Behavior and Consequences on Wildlife

On average, the cats spent about 2.5 h outside per day. Though often described as a nocturnal species, they were mostly active during the day while outdoors, being active for approximately 2 h per day, which corresponds to 87.7% of their time spent outside (combination of exploration, observation, and predation categories). These durations seem quite short but are probably underestimated as the total time spent outside could not be determined (see the description of behavioral data processing above). Nevertheless, the durations are substantial in terms of the landscape of fear generated for prey, especially when cats are active and thus easily detectable. The numerous species visible on the videos, from insects to birds, confirm this idea.

Predation behavior was highly variable between individuals. Indeed, for some individuals, no predation event was recorded during the sampling period, while others hunted up to nine times per day. For the hunters, important intraindividual variations were also observed, including days without predation attempts. With 31 prey caught in 10 days by 9 out of 13 monitored individuals, the impact of one domestic cat on wildlife during the day can be considered low. However, the sublethal effects induced by the landscape of fear should be considered, as well as the density and number of individuals, which can be high in suburban areas and pressure wildlife (Liberg et al. 2000; Woods et al. 2003; Pavisse and Vangeluwe 2019). In addition, most of the existing restrictive management measures focus on nighttime and thus exclude daytime predation (Cecchetti, Crowley, and McDonald 2021).

The study also demonstrated that the use of animal‐borne cameras is more reliable than the prey‐report method used in most studies on cat predation (Loyd et al. 2013), as only one out of the 31 prey caught was brought home; furthermore, failed predation attempts (29 events) could not be assessed otherwise. The prey‐report method is also biased in terms of the diversity of prey returned (Krauze‐Gryz et al. 2017) and can be limited by the ability of owners to identify the prey due to their lack of expertise (Lockwood et al. 2025) or the condition of the prey at the time of discovery. Therefore, the use of animal‐borne cameras represents significant progress to better estimate the predation of domestic cats by quantifying the number of prey caught, probably severely underestimated with the prey‐report method. In addition, this type of monitoring allows participants and scientists to more accurately identify prey as they are visible on camera in an almost pristine condition.

### 4.4 Effect of Cat Personality on Predation Behavior

The results of models (i) and (ii) revealed that predation behavior was partially influenced by the personality of the cat. Cats with high levels of agreeableness and neuroticism indeed spent significantly less time hunting. Model (ii) had a low explanatory power due to the small number of predation events but showed the same significant effect of agreeableness, which was associated with fewer predation events. Neuroticism wasn't significantly associated with fewer predation events (*p* = 0.087) due to the low explanatory power of model (ii). The mitigating effect of neuroticism on predation in domestic cats has already been shown by (Cecchetti, Crowley, McDonald, and McDonald 2022). Considering that neurotic individuals have smaller ranges, they probably encounter fewer prey, with their fearful personality making them more vigilant when outside and less focused on predation. The same study also showed that extraverted cats are more likely to hunt, though this effect wasn't significant in our study (*p* = 0.075 in model (i)) due to our small sample size. As they are more explorative individuals (perhaps with wider ranges), they encounter more prey and are more likely to hunt them, suggesting that predation may be stimulating for them. Playing with these individuals could thus effectively reduce predation (Cecchetti, Crowley, Goodwin, and McDonald 2021). Agreeable cats are less inclined to hunt, partly due to their reduced range size but also because they perhaps tend to spend more time with their owners who give them more attention through more frequent play sessions or more food like treats.

For both models, the identity of the cat coded as a random factor had a non‐negligible effect, highlighting the importance of other determinants in the shaping of an individual's predation behavior. Biological factors (age, sex), habitat type (open land, highly vegetated area), and owner's behavior (feeding, hygiene, play) have been shown to have an influence on predation and might interact with personality as well (Foreman‐Worsley et al. 2021).

### 4.5 Prospects

Our study paves the way for further investigations into the influence of individual personality on the predation behavior of domestic cats. Although our results are promising, this study has several limitations. Our small sample size (determined by the number of devices we could acquire) did not allow us to compare the influence of personality and other predictors on roaming and predation behaviors. Technological constraints also limited our study: the devices were not easy for owners to use and sometimes encountered software errors, leading to data loss and variability in the total duration of data collection among cats. More robust and user‐friendly devices would reduce data loss, facilitate cat recruitment, and enable longer monitoring periods by minimizing the burden on volunteer owners. A straightforward improvement would be to incorporate a GPS transmitter to help locate and retrieve devices lost due to the collars' breakaway safety mechanism. Future advances in device miniaturization will further expand monitoring possibilities. For example, fitting two different lenses for day and night vision would provide a more comprehensive understanding of predation behavior, as day and night variations in predation behavior are probably important. Indeed, although cats' ranges differ little between day and night (Hanmer et al. 2017), differences in prey availability likely drive changes in predation behavior (Woods et al. 2003).

Another limitation is that the results of this study cannot be generalized to all contexts, as the cats in our study all had similar living conditions. This low variability was intentional, as it helped minimize the effects of confounding factors and was partly ensured by the small size of the study area. It was further confirmed through the habits assessment (feeding, play, hygiene…) conducted alongside the personality questionnaire. Future studies should therefore investigate a broader range of contexts, for example by examining variations along the urbanization gradient. As range size (Hanmer et al. 2017; Kays et al. 2020; Cordonnier et al. 2022) and prey diversity (McGregor et al. 2015; Cordonnier et al. 2022; Herrera et al. 2022) depend on the environment, cats could display different behaviors in accordance with their personality. Seasonal variations could also be explored. Although the home range size is unlikely to change between seasons (Thomas et al. 2014; Philippe‐Lesaffre et al. 2024), predation behavior substantially varies because of changes in prey availability and meteorological conditions (temperature, humidity) (Castañeda et al. 2023) to which cats may respond differently depending on their personality. When completing the questionnaire, many owners reported less activity and less time spent outdoors during cold months, which supports this hypothesis. Though the macroenvironment was similar for all cats, differences in the microenvironment (presence of refuge areas, microhabitats attracting different wild species…) could not be assessed and could have an impact on the behavior.

Our study also focuses on owned cats matched with precise criteria (see study area and cat recruitment). Other types of cats, especially stray cats, behave differently and can have larger ranges and hunt more (Cordonnier et al. 2022). These populations, which are difficult to manage (Cecchetti, Crowley, and McDonald 2021), can be numerous (around 250,000 in the UK; McDonald and Skillings 2021 and at least 11 million in France; Eichstadt 2020) and cause more severe damage than owned cats.

The development of artificial intelligence could also accelerate the currently time‐consuming treatment of video data using behavior recognition models. These tools could also be used to analyze videos extracted from social media, where cat videos are uploaded in astonishing quantities (Leighton and Serieys 2024), and animal‐borne camera recordings are increasingly numerous as these devices become relatively financially accessible for private individuals. A global participative science initiative could also be designed, with cat owners uploading animal‐borne videos of their cats to the internet after completing a questionnaire on habits and a personality test for their cats. Beyond the scientific interest of a massive video database to more accurately estimate cat predation pressure according to the season, environment, and individual cat covariables, this initiative would also spread awareness among cat owners about the magnitude of predation on wildlife and facilitate the adoption of responsible management measures if required (Cecchetti, Crowley, and McDonald 2021).

## 5 Conclusion

Our study surveyed 23 domestic cats in a French suburban area using combined GPS and animal‐borne camera devices. The personality of these individuals assessed through an owner‐completed questionnaire linked their space use and predation behavior. Our results highlight the influence of the individual's personality on its range size, the time spent hunting, and the number of predation events. Agreeable and neurotic cats have smaller ranges and are less inclined to hunt than other individuals. The innovative use of animal‐borne cameras offered a deeper insight into the outside behavior of domestic cats during the day, with 2 h of activity per day on average. Compared to the prey‐report method, it is also a more reliable tool to assess predation events. Further investigations, with the enrolment of more participants facilitated by the progress of miniaturized technologies, will help implement personalized mitigation measures that account for the cat's personality and other proven drivers of predation (biological factors, owner practices, etc.).

## Author Contributions

**Bastien Berrand:** data curation (lead), formal analysis (lead), investigation (lead), methodology (equal), visualization (lead), writing – original draft (lead), writing – review and editing (equal). **Emmanuelle Baudry:** conceptualization (equal), funding acquisition (equal), methodology (equal), project administration (equal), resources (equal), supervision (equal), writing – review and editing (equal). **Elsa Bonnaud:** conceptualization (equal), funding acquisition (equal), methodology (equal), project administration (equal), resources (equal), supervision (equal), writing – review and editing (equal).

## Funding

This work was supported by Ecologie Société Evolution, Cross‐team project (RETIE), Agence Nationale de la Recherche, Top_Pred, Garland, and Université Paris‐Saclay, Labex BASC.

## Conflicts of Interest

The authors declare no conflicts of interest.

## Supporting information


**Appendix S1:** Detailed ethogram for video data analysis.
**Appendix S2:** Variable table.
**Appendix S3:** Detailed description of the recorded prey species.

## Data Availability

The dataset associated with this manuscript has been deposited on dryad (https://doi.org/10.5061/dryad.jm63xsjrs). Raw data containing personal information (video data, full GPS tracks) is not publicly available due to privacy constraints.
