# Supplementary material for: Personality Matters: Owner‐Ascribed Personality Predicts Range Size Tendencies and Predation Behavior in Domestic Cats
Source: Ecol Evol. 2026 Apr 29;16(5):e73520. doi: 10.1002/ece3.73520 (PMC13126088; doi:10.1002/ece3.73520)
Supplement: Supplementary file 1 — Appendix S1: Detailed ethogram for video data analysis. Appendix S2: Variable table. Appendix S3: Detailed description of the recorded prey species. [file ECE3-16-e73520-s001.docx]

**Appendix I – Detailed ethogram for video data analysis**

| **Behavioral event name** | **Category** | **Description** |
| --- | --- | --- |
| MOVE | Exploration | Cat walking or running without a visible purpose |
| DRINK | Exploration | Cat drinking from any source of water |
| FORAGE | Exploration | Cat sniffing, scratching, or digging |
| OBSERVE | Observation | Cat immobile but awake, with head movements or short precise focus |
| HUNT | Predation | Cat running after prey |
| CARRY | Predation | Cat carrying prey |
| EAT | Predation | Cat consuming prey |
| REST | Inactivity | Cat immobile and not awake, no movement at all |

**Appendix II – Variable table**

| **Variable name** | **Description** | **Model** |
| --- | --- | --- |
| aKDEc50 | Core range in hectares | aKDEc50 model |
| aKDEc95 | Full range in hectares | aKDEc95 model |
| Predation time | Time spent hunting in seconds | Model i |
| Predation events | Amount of predation events (count) | Model ii |
| Personality traits (agreeableness, dominance, extraversion, neuroticism) | Personality score calculated with exploratory factor analysis | All models |
| Time | Total duration of the video | Models i and ii |
| Cat identity | Unique cat number | Models i and ii |

**Appendix III – Detailed description of the recorded prey species**

| **Proposed identification** | **Taxonomic level** | **Certainty** |
| --- | --- | --- |
| European peacock – *Aglais io* | Species | Certain |
| Eurasian magpie – *Pica pica* | Species | Certain |
| Buff-tailed bumblebee – *Bombus terrestris* | Species | Certain |
| Common wall lizard – *Podacris muralis* | Species | Most probable |
| Common wall lizard – *Podacris muralis* | Species | Most probable |
| Common wall lizard – *Podacris muralis* | Species | Certain |
| Beetle | Order | Unsure |
| Lepidoptera | Order | Certain |
| Hymenoptera | Order | Most probable |
| House sparrow – *Passer domesticus* | Species | Certain |
| Grove snail – *Cepaea nemoralis* | Species | Certain |
| Insect | Class | Most probable |
| Wood mouse – *Apodemus sylvaticus* | Species | Certain |
| Wood mouse – *Apodemus sylvaticus* | Species | Certain |
| *Microtus sp* | Genus | Certain |
| Common wood pigeon – *Columba palumbus* | Species | Certain |
| Lepidoptera | Order | Certain |
| Soricidae | Family | Certain |
| Lepidoptera | Order | Certain |
| *Microtus sp* | Genus | Certain |
| *Microtus sp* | Genus | Certain |
| *Microtus sp* | Genus | Certain |
| Rodent | Order | Most probable |
| Wood mouse – *Apodemus sylvaticus* | Species | Certain |
| Wood mouse – *Apodemus sylvaticus* | Species | Certain |
| Wood mouse – *Apodemus sylvaticus* | Species | Certain |
| *Microtus sp* | Genus | Certain |
| Wood mouse – *Apodemus sylvaticus* | Species | Most probable |
| *Microtus sp* | Genus | Certain |
| Wood mouse – *Apodemus sylvaticus* | Species | Certain |
| *Microtus sp* | Genus | Certain |
| Rodent | Order | Most probable |
| *Microtus sp* | Genus | Certain |
| *Microtus sp* | Genus | Certain |
| *Microtus sp* | Genus | Certain |
| Wood mouse – *Apodemus sylvaticus* | Species | Certain |
| *Microtus sp* | Genus | Certain |
| Wood mouse – *Apodemus sylvaticus* | Species | Certain |
| *Microtus sp* | Genus | Certain |
| Wood mouse – *Apodemus sylvaticus* | Species | Certain |
| *Microtus sp* | Genus | Certain |
| *Microtus sp* | Genus | Certain |
